# Relocation Frequency and Loneliness Among Older Evacuees: Associations with Perceived Community Resilience

**DOI:** 10.3390/healthcare14213755

**Published:** 2026-11-04

**Authors:** Adi Vitman Schorr, Aviva Zrihan Weitzman

**Affiliations:** School of Social Work, Tel Hai University of Kiryat Shmona in the Galilee, Upper Galilee 1220800, Israel; avivaw@telhai.ac.il

**Keywords:** evacuation, loneliness, community resilience, relocation frequency, older adults

## Abstract

**Highlights:**

**What are the main findings?**

• Examines relocation frequency using its categorical response structure rather than treating unequal ordinal categories as a continuous score.
• Finds differences in loneliness across relocation-frequency categories after demographic adjustment, but no monotonic dose–response pattern and attenuation after broader adjustment for depressive symptoms, health, and income.
• Shows that perceived community resilience is associated with lower loneliness, while the cross-sectional data do not support a mediation interpretation.

**What are the implications of the main findings?**

• Evacuation services should consider relocation histories as one component of psychosocial assessment while recognizing that loneliness is also strongly related to individual and socioeconomic factors.
• Gerontological practice should screen older evacuees for loneliness and relevant vulnerabilities, including depressive symptoms and lack of a partner, rather than using relocation frequency alone as a risk marker.
• Future research should use longitudinal designs and multidimensional measures of relocation experiences, community continuity, service access, and loneliness in displaced older populations.

**Abstract:**

Background and Objectives: Older adults evacuated during armed conflict may experience repeated residential moves in addition to the initial evacuation. Because the number of relocations in this study was recorded in unequal ordinal categories, the present analysis treats relocation frequency as a categorical exposure rather than as a continuous measure of a broader construct of relocation instability. This study examined post-return associations between self-reported relocation-frequency category, perceived community resilience, and loneliness among adults aged 50 years and older following wartime evacuation. Research Design and Methods: The analytic sample included 109 evacuees (eligibility ≥ 50 years; observed age range 54–89 years) from a northern Israeli community who had returned to their home community following evacuation during the Israel–Hamas War. Loneliness was measured using the 11-item Hebrew version of the De Jong Gierveld Loneliness Scale and perceived community resilience using the brief Conjoint Community Resiliency Assessment Measure (CCRAM-10). Relocation frequency was assessed with a single study-specific self-report item and analyzed in three categories (one relocation, two to three relocations, or four or more relocations); because the questionnaire did not explicitly define which transitions to count, the categories are interpreted as self-reported. Multivariable linear regression used indicator coding with one relocation as the reference category and adjusted for age, gender, and partnership status; sensitivity analyses additionally adjusted for depressive symptoms, current physical health, and household income. Results: Loneliness differed across relocation categories in the demographic-adjusted model (overall F(2,88) = 7.40, *p* = 0.001), but the pattern was not monotonic. Participants reporting two to three or four or more relocations had higher adjusted loneliness than the one-relocation group. Perceived community resilience was associated with lower loneliness (B = −0.312, *p* = 0.002), but relocation category was not significantly associated with community resilience (overall *p* = 0.187). In a same-sample comparison (*n* = 76), the relocation coefficients decreased after adding depressive symptoms, current physical health, and household income, and the overall relocation category term was not statistically significant in the extended model (*p* = 0.298). Discussion and Implications: Self-reported relocation-frequency category may mark heterogeneity in post-return experiences following evacuation, but the observed association with loneliness was sensitive to adjustment for additional psychosocial and socioeconomic factors. Cross-sectional data and a single-item relocation measure do not support causal or mediation conclusions. Longitudinal studies using multidimensional measures of relocation experiences are needed.

## 1. Introduction

Migration refers to the movement of people from one geographical area to another and may be voluntary or forced [1]. Forced migration encompasses involuntary movement arising from threats to safety or security. Within this broader context, displacement refers to being compelled to leave one’s usual place of residence, whereas evacuation refers to an organized relocation to a safer location, typically intended to be temporary and undertaken with an expectation of return [1]. In the present study, evacuation represents a government-directed form of forced movement in the context of armed conflict. However, evacuation may involve not only an initial relocation but also repeated changes in residence between temporary locations over time [2].

From an environmental gerontology perspective, evacuation can be interpreted in relation to ageing in place, which emphasizes continuity between older adults and their physical and social environments. Environmental gerontology emphasizes the dynamic relationship between older adults and their physical and social environments, with well-being shaped in part by the fit between individual competencies and needs and environmental demands and resources [3]. Ageing in place extends beyond remaining in the same dwelling; it encompasses continuity, familiarity, autonomy, social relationships, and attachment to home and community [4,5]. Repeated relocations may disrupt these person–environment relations by repeatedly altering the physical setting, daily routines, social surroundings, and access to familiar community resources. These environmental gerontology concepts provide the theoretical context for this study but were not measured directly. The empirical model therefore focuses specifically on the association between relocation frequency, perceived community resilience, and loneliness.

According to a scoping review on older adults in times of armed conflict [6], conflict situations exacerbate existing inequalities affecting older persons, including the lack of prioritization in policy and evacuation efforts despite their heightened vulnerability [7]. In fact, older adults are often not evacuated as a matter of policy, leaving them exposed to ongoing threats [6,7]. Research on their evacuation experiences during such events remains limited. This shortcoming is particularly significant given recent findings from the Israel–Hamas War, where a comparison between evacuated and non-evacuated older adults [8] revealed that evacuees reported lower levels of mental wellbeing and lower subjective health. Among those evacuated, the security threat was identified as a strong predictor of their diminished sense of control [9], underscoring the urgent need to better understand and address the unique challenges older adults face during forced displacement.

In Israel, the Israel–Hamas War created a large-scale evacuation of residents from northern border communities that included older adults, making this context especially relevant for examining the psychosocial implications of evacuation [10]. Within this context, older adults represent a particularly important population. Though they may be more vulnerable due to chronic health conditions, reduced functional capacity, and the cumulative impact of stressful life events [11], their life experience may enhance their ability to cope with adversity and stress [12,13].

Loneliness and community resilience are central to understanding the psychosocial consequences of evacuation among older adults [14,15,16]. Accordingly, the present study examines whether post-return loneliness differs across self-reported relocation-frequency categories experienced during the evacuation period and whether perceived community resilience is associated with loneliness after accounting for relocation frequency and background characteristics.

## 2. Literature Review

**Loneliness** is defined by Perlman and Peplau [14] as an unpleasant experience that arises when a person’s network of social relationships is deficient in some important way. Loneliness and social isolation are often used interchangeably, but they are two distinct concepts. Social isolation refers to objective, quantifiable social contacts and interactions, while loneliness refers to the experience of a perceived discrepancy between desired and actual social relationships [17,18]. Older adults are at higher risk of experiencing both loneliness and social isolation [15]. Loneliness is a central factor contributing to numerous negative mental health outcomes in older adults, including anxiety, cognitive disorders, and depression [19,20].

Although research on loneliness in older adults in the context of war seems to be limited, numerous studies explored loneliness among older adults during the COVID-19 pandemic. In a systematic review and meta-analysis including 45 studies, Su et al. [21] reported that older adults experienced significantly higher levels of loneliness in studies conducted after the first three months of the COVID-19 pandemic compared with those conducted during the initial three months. Similarly, in the context of the Israel–Hamas War, loneliness has been associated with suicidal ideation among Israeli civilians [22], while broader emotional distress has also been documented among Israeli civilians, including evacuees [23]. In the context of Israeli older adults, individuals displaced from their homes due to the conflict have reported higher levels of loneliness compared to those who did not live in a war zone and were not evacuated from their homes [9]. Evidence from another study conducted in Israel indicated that, among older adults, loneliness mediates the relationship between exposure to terror attacks and psychological distress [24]. These findings highlight the potential importance of examining how characteristics of evacuation, such as relocation frequency, may be associated with loneliness among older adults.

Displacement during wartime: Relocation frequency is one observable feature of the post-evacuation residential experience. Previous research has operationalized residential instability using the number of residential moves made over time, while post-disaster housing research has similarly examined frequent moves and prolonged transitions before stable housing is regained [25,26]. However, the number of moves alone does not constitute a multidimensional measure of relocation instability. It does not directly assess duration of displacement, housing insecurity, voluntariness, distance from home, accommodation quality, uncertainty, place attachment, person–environment fit, continuity, or social embeddedness. The present study therefore uses the narrower term relocation frequency and analyzes relocation frequency categorically rather than assuming equal intervals between them. Environmental gerontology concepts are used as an interpretive context for why repeated changes in residence may matter in later life, not as measured components of the relocation-frequency variable. Following the outbreak of the Israel–Hamas War in October 2023, the Israeli government implemented its “guest hotel” policy, under which thousands of residents of northern border communities were required to leave their homes and relocate to designated facilities [10]. Although the evacuation was intended as a protective measure, the relocation arrangements were often temporary, resulting in repeated changes in residence during the ongoing conflict.

Such instability may be particularly consequential for older adults, who are at increased risk of mental health challenges including depression, anxiety, stress [27,28] and loneliness [18], especially in wartime, when social support may be limited [6]. Displacement may further exacerbate these risks by disrupting attachment to place, a key protective factor that supports continuity and emotional stability in later life [29,30].

Empirical evidence from the Israel–Hamas War has indicated that displacement is associated with reduced autonomy, disrupted routines and challenges to mental well-being across populations [8,31,32] while also highlighting the importance of social and cultural bonds in maintaining resilience [8].

Beyond displacement itself, repeated relocations may be associated with additional disruption of access to services and social ties [2,33]. Frequent residential moves have been associated with poorer mental health outcomes in disaster contexts [34,35], and multiple displacements have been associated with reduced access to essential resources [36]. These findings provide a rationale for examining relocation frequency while avoiding the assumption that frequency alone captures the broader psychosocial construct of relocation instability.

Accordingly, relocation frequency may be associated with psychological outcomes, although social and individual characteristics may account for part of this association [37]. Perceived community resilience may also be relevant because it reflects residents’ perceptions of collective resources, trust, preparedness, and support. The present study therefore examined relocation-frequency categories and perceived community resilience as concurrent correlates of loneliness; no temporal or mediation model was specified. Most previous research has compared displaced with non-displaced populations; less is known about heterogeneity among evacuees who experience different numbers of residential moves. The present study addresses this gap by examining relocation-frequency categories within an evacuee-only sample.

Community and community resilience: Community provides a social and symbolic context for belonging, cohesion, trust, identity, and access to support [38,39,40,41,42,43].

Two central aspects of community are social capital and social cohesion. Social capital refers to resources embedded in social networks and is reflected in trust, reciprocity, shared norms, and access to support from family, friends, and the wider community [41,42,43]. In times of crisis, social cohesion, solidarity, shared identity, and place attachment may support mutual support networks and strengthen perceived personal and collective control [30,44,45,46,47,48].

Community resilience is a crucial factor in a community’s ability to cope with disasters and crises. It reflects the joint capacity of residents and institutions to respond to unexpected events, adapt to changing conditions, and maintain functioning after adversity [15,16,49,50]. Community resilience is shaped by several forms of capital, including symbolic, social, organizational, material and human capital, as well as by social cohesion, optimism, leadership, community involvement and trust [51,52,53,54,55].

Community resilience is reflected both objectively, when the community provides an actual response to residents’ needs, and subjectively, when individuals perceive community resources as available to them [50,56].

Community resilience is positively associated with individual resilience, lower stress and psychological distress, and higher levels of post-traumatic recovery [57,58].

Among older adults, community engagement, agency, leadership, and connection to place may support perceived community resilience [59]. In repeated evacuation-related relocations, disrupted social ties and access to community resources may be relevant to loneliness, although these mechanisms were not measured directly.

### 2.1. Study Objective

The objective of this study was to examine whether loneliness differed across the three reported relocation-frequency categories among older adults evacuated from the northern periphery during the Israel–Hamas War. A second objective was to examine whether perceived community resilience was associated with loneliness after accounting for relocation-frequency category and background characteristics. Additional sensitivity analyses assessed whether the relocation-frequency association persisted after adjustment for depressive symptoms, current physical health, and household income.

### 2.2. Hypotheses

After controlling for background characteristics, we expected that: (1) loneliness would differ across relocation-frequency categories, with the higher-frequency categories expected to show higher loneliness relative to the one-relocation category; and (2) lower perceived community resilience would be associated with higher loneliness after accounting for relocation-frequency category. Because the response categories represent unequal ranges of moves, no linear 1-to-4 trend was assumed.

Age, gender, and partnership status were included as prespecified demographic covariates. To examine the sensitivity of the findings to additional covariates, depressive symptoms, current physical health, and household income were added in an extended model; education was examined in an additional complete-case sensitivity analysis.

## 3. Method

### 3.1. Research Design

This study employed a cross-sectional survey design among older adults evacuated from the northern Israeli periphery during the Israel–Hamas War. Ethical approval was granted on 22 April 2025, and data collection began on 25 April 2025; no participant data were collected before ethical approval. Data were collected through June 2025, after participants had returned to their hometown following evacuation.

### 3.2. Participants

The broader survey included 142 participants recruited in one northern Israeli community. The present analytic sample included 109 participants who reported at least one evacuation-related relocation; 26 participants who selected the no-relocation response and seven with missing relocation-frequency data were excluded from the present analysis. A separate item on evacuation accommodation showed that all 26 participants selecting the no-relocation response had evacuated: 13 reported evacuations to a hotel, 2 to an independently rented apartment, 8 to relatives or friends, and 3 to another arrangement. Their “no relocation” response therefore did not indicate non-evacuation and was treated as ambiguous with respect to the intended relocation count. Eligibility for the present analysis required age 50 years or older, consistent with the SHARE sampling frame [60], residence in the study community, at least one self-reported evacuation-related relocation, and return to the hometown by the time of data collection. Accordingly, the target population for inference is returnees to this community rather than all older evacuees affected by the conflict. Participants provided informed consent and completed the Hebrew questionnaire. The study records available for the present analysis do not document a separate formal cognitive-screening procedure. The term “older adults” is used here as a descriptive study-population label rather than as a universal age cutoff. The ≥50-year threshold was selected to align with the SHARE 50+ framework and because this study focused on later-life experiences that may differ from those of younger adults. No a priori sample-size or power calculation was conducted; the analytic sample comprised all eligible participants who consented and reported at least one evacuation-related relocation during the data-collection period. The analytic sample consisted of a majority of women (84), and most participants with partnership-status data had a partner. The observed age range was 54–89 years (M = 69.5, SD = 7.0). Because the sample included participants younger than 65 years, the primary demographic-adjusted model was also repeated in a sensitivity analysis restricted to participants aged 65 years or older.

### 3.3. Procedure

Following approval from the university’s ethics board, participants were recruited through two complementary approaches: some were approached directly in public spaces in the community, while others were contacted using contact details provided by the local authority. In both recruitment routes, individuals who expressed interest in participating received the same link to the online questionnaire and completed the questionnaire independently. Contact information was used only for recruitment and distribution of the survey link and was not linked to questionnaire responses. No identifying information was collected as part of the questionnaire, and survey responses were therefore anonymous. Participation was voluntary, and informed consent was obtained before completion of the questionnaire. Participants could decline participation or discontinue the questionnaire at any time before submission without consequence. A complete denominator of all potentially eligible residents approached for participation was not available in the study records; therefore, a conventional response rate could not be calculated.

### 3.4. Measurements

#### 3.4.1. Loneliness

Loneliness was measured using the 11-item De Jong Gierveld Loneliness Scale originally developed by De Jong Gierveld and Kamphuis [61] and subsequently validated in Hebrew among older adults in Israel [62]. The scale contains six negatively phrased items (items 2, 3, 5, 6, 9, and 10; e.g., “I experience a general sense of emptiness”) and five positively phrased items (items 1, 4, 7, 8, and 11; e.g., “There are plenty of people I can rely on when I have problems”). The Hebrew questionnaire used five response options ranging from 1 (“definitely yes”) to 5 (“definitely no”). In the present scoring, the six negatively worded items were reverse-scored and all 11 item scores were summed so that higher total scores indicated greater loneliness. The theoretical range was 11–55. The observed range in the present sample was 11–45, and internal consistency was good (Cronbach’s α = 0.84). The five-category response format and the Hebrew version have been used and psychometrically evaluated in Israeli older-adult samples [62].

#### 3.4.2. Number of Relocations

Relocation frequency was measured with a single self-report item constructed for the current study. The intended operational definition counted the initial evacuation from the participant’s home to another location as the first relocation and each subsequent move between temporary residential locations as an additional relocation; return to the permanent home was not intended as an additional relocation. Participants were asked: “If you are an evacuee, how many relocations have you undergone since the beginning of the war?” Responses were given on a four-category ordinal scale: (1) no relocations, currently in the same place; (2) one relocation; (3) two to three relocations; and (4) four or more relocations. The questionnaire did not explicitly state this counting rule or separately instruct participants whether to count the initial evacuation, moves between temporary accommodations, or the return home. Consequently, some participants may have interpreted what constituted a relocation differently, introducing potential exposure misclassification. For the present evacuee-only analysis, participants who selected “no relocations, currently in the same place” were excluded; relocation frequency was analyzed in three categories (one relocation, two to three relocations, and four or more relocations) using indicator coding with one relocation as the reference category. A separate evacuation-accommodation item indicated that all 26 participants who selected “no relocations” had in fact evacuated. This response therefore could not be interpreted as evidence of non-evacuation and may reflect a different understanding of what counted as a relocation. Because their number of moves could not be classified consistently with the intended counting rule, they were excluded from the primary three-category analysis and retained as a fourth response category in a sensitivity analysis. Previous research has used the number or frequency of moves as one indicator of residential or housing instability [25,26], but the present item is interpreted only as a self-reported relocation-frequency category.

Because relocation frequency was assessed using a single item, internal consistency and other psychometric properties could not be evaluated. The item does not measure the broader multidimensional experience of relocation or housing instability and should not be interpreted as a validated measure of disruption in place attachment, person–environment fit, continuity, or social embeddedness.

#### 3.4.3. Community Resilience

Perceived community resilience was measured using the brief Conjoint Community Resiliency Assessment Measure (CCRAM-10) [52]. The questionnaire instructions explicitly asked participants to rate the statements with reference to their permanent place of residence. Thus, the measure reflects participants’ current perception of the resilience of their permanent/home community at the time of the survey; it does not measure the resilience of a temporary evacuation setting or community conditions experienced during each relocation. The scale includes 10 items (e.g., “The local government of my community functions well” and “I can count on people in my community to help me in a crisis situation”), rated from 1 (disagree) to 5 (very strongly agree). Item scores were summed, yielding a theoretical range of 10–50, with higher scores indicating greater perceived community resilience. Internal consistency in the present sample was good (Cronbach’s α = 0.84).

#### 3.4.4. Additional Covariates for Sensitivity Analyses

Depressive symptoms were assessed using the 15-item Geriatric Depression Scale (GDS-15) [63]. Items were scored 0 or 1 and summed so that higher scores indicated more depressive symptoms; internal consistency in the present sample was α = 0.74. Current physical health was assessed with a single item rated from 1 (very poor) to 5 (very good). Household income was reported on a five-category scale ranging from much below to much above the stated national average. Years of education were also collected and were examined in an additional complete-case sensitivity analysis because of greater item nonresponse.

### 3.5. Data Analyses

Univariate analyses described the sample and study variables, and Cronbach’s alpha was calculated for multi-item measures. The analytic sample was restricted to participants reporting at least one evacuation-related relocation. Because of item nonresponse, descriptive and bivariate analyses used available cases, whereas each regression used complete cases for the variables included in that model; no values were imputed. Complete-case regression was used in the absence of a prespecified imputation model. No formal test of the missing-data mechanism was conducted; therefore, selection bias related to missingness cannot be excluded, and model-specific N is reported. The complete-case approach was retained rather than introducing a post hoc imputation model; the observed difference in partnership status between included and excluded cases is therefore treated as a potential source of selection bias. All inferential tests were two-sided. Bivariate associations with loneliness were examined using Pearson’s correlations for age and community resilience, independent-sample *t* tests for gender and partnership status, and one-way analysis of variance for the relocation-frequency category. In the primary multivariable analysis, relocation frequency was entered as a categorical predictor using two indicator variables, with one relocation as the reference category; it was not treated as a continuous score. Model 1 adjusted for age, gender, and partnership status. Model 2 additionally included perceived community resilience to estimate its concurrent cross-sectional association with loneliness while retaining relocation category in the model. A separate regression examined whether relocation category was associated with perceived community resilience. The extended sensitivity model was based on Model 1 and added depressive symptoms, current physical health, and household income; perceived community resilience was not included. Because these covariates may be related to loneliness and evacuation experiences and were measured after return, they could represent pre-existing confounders, concurrent correlates, or possible consequences of evacuation and relocation. This model was therefore interpreted as a sensitivity analysis rather than as estimating an independent causal effect. To distinguish covariate adjustment from changes in sample composition, Model 1 was also re-estimated in the same 76 participants included in the extended model, and the relocation coefficients and 95% confidence intervals were compared across the two specifications. Because the separate evacuation-accommodation item showed that all 26 respondents selecting “no relocation” had nevertheless evacuated, an additional sensitivity analysis retained this response as a fourth relocation category rather than excluding these participants. Household income was entered as the ordered five-category variable; because equal spacing across categories cannot be assumed, an additional sensitivity analysis treated income categorically. A further complete-case model added years of education. To examine whether the results depended on the broad age-50-plus recruitment framework, Model 1 was repeated among participants aged 65 years or older. The single participant who selected a gender category other than man or woman was retained in descriptive analyses but was not included in regression models requiring binary gender coding. Regression diagnostics were examined using residual plots, collinearity diagnostics, and Cook’s distance. Across the multivariable models, variance inflation factors were below 1.7 and Cook’s distances did not exceed 0.11; residual inspection did not indicate marked departures from linearity or residual normality. Breusch–Pagan testing suggested mild heteroskedasticity in Model 1 (*p* = 0.046) but not in Model 2 or the extended model (*p* = 0.097 and *p* = 0.204, respectively); HC3 heteroskedasticity-robust standard errors were therefore examined in sensitivity analyses. All statistical analyses were conducted using Python (version 3.13.5), with statsmodels (version 0.14.6) and SciPy (version 1.17.0). All analyses are associational; no mediation or causal temporal ordering was tested.

## 4. Results

The analytic sample consisted of 109 evacuees, including 84 women, 24 men, and one participant who selected another gender category. Among participants with partnership-status data, 65 were classified as having a partner and 42 as not having a partner. Age was available for 102 participants and ranged from 54 to 89 years (M = 69.5, SD = 7.0).

Loneliness scores were available for 102 participants (M = 25.1, SD = 8.0; range 11–45), and community resilience scores were available for 96 participants (M = 34.3, SD = 7.8; range 17–50). Relocation-frequency categories in the 109-participant analytic sample were: one relocation, *n* = 32 (29.4%); two to three relocations, *n* = 58 (53.2%); and four or more relocations, *n* = 19 (17.4%). Because these categories do not represent equal numerical intervals, no mean relocation score is reported (Table 1).

Missing observations in the 109-participant analytic sample were: age, *n* = 7; partnership status, *n* = 2; loneliness, *n* = 7; community resilience, *n* = 13; depressive symptoms, *n* = 15; current physical health, *n* = 4; household income, *n* = 14; and usable numeric years of education, *n* = 29. Model 1 included 94 participants. In an exploratory comparison, participants included in Model 1 were more likely to have a partner than those excluded (64.9% vs. 30.8% among those with partnership-status data; Fisher’s exact *p* = 0.031); no statistically significant differences were observed for age, loneliness, relocation category, community resilience, depressive symptoms, physical health, household income, or binary gender.

Descriptive characteristics also varied across relocation-frequency categories. For the one-, two-to-three-, and four-or-more-relocation groups, respectively, mean age was 71.5, 69.5, and 66.4 years; 62.5%, 49.1%, and 94.4% had a partner; mean GDS-15 scores were 2.7, 4.4, and 4.0; mean physical-health scores were 3.4, 3.1, and 3.3; median household-income category was 3 in all three groups; mean community-resilience scores were 36.2, 33.5, and 33.1; and mean loneliness scores were 21.5, 27.1, and 25.1.

Table 2 presents the bivariate associations with loneliness. Gender was not significantly associated with loneliness, whereas older age and not having a partner were associated with higher loneliness. Participants with a partner reported lower loneliness (M = 22.7, SD = 6.9) than participants without a partner (M = 28.9, SD = 8.5). Loneliness differed across relocation-frequency categories, F(2,99) = 5.07, *p* = 0.008, η^2^ = 0.093. The unadjusted group means were 21.5 for one relocation, 27.1 for two to three relocations, and 25.1 for four or more relocations, indicating that the pattern was not monotonic. Higher perceived community resilience was associated with lower loneliness (r = −0.39, *p* < 0.001). The relocation groups were uneven, and the ≥4-relocation group was relatively small (*n* = 19); category-specific estimates should therefore be interpreted with corresponding uncertainty.

### Multivariable Analyses

In the demographic-adjusted model (Model 1; *n* = 94), the full model was significant, F(5,88) = 7.36, *p* < 0.001, R^2^ = 0.295. Relocation-frequency category was associated with loneliness overall, F(2,88) = 7.40, *p* = 0.001. Relative to participants reporting one relocation, those reporting two to three relocations (B = 5.959, *p* < 0.001) and four or more relocations (B = 6.499, *p* = 0.005) had higher adjusted loneliness. Older age and not having a partner were also associated with higher loneliness; gender was not. A sensitivity analysis restricted to participants aged 65 years or older (*n* = 75) yielded the same overall conclusion: relocation category remained significant, F(2,69) = 5.27, *p* = 0.007. In this age-restricted model, both the two-to-three-relocation category (B = 5.546, *p* = 0.003) and the four-or-more category (B = 5.635, *p* = 0.033) were higher than the one-relocation category. When perceived community resilience was added (Model 2; *n* = 86), the full model was significant, F(6,79) = 9.07, *p* < 0.001, R^2^ = 0.408. Higher community resilience was associated with lower loneliness (B = −0.312, *p* = 0.002), and the overall relocation-category term remained significant, F(2,79) = 7.19, *p* = 0.001. In a separate model with community resilience as the outcome (*n* = 87), the full model was not statistically significant, F(5,81) = 1.66, *p* = 0.155, R^2^ = 0.093, and the overall association with relocation-frequency category was not significant, F(2,81) = 1.71, *p* = 0.187. These cross-sectional results do not support treating community resilience as a mediator of relocation frequency. HC3 robust-standard-error sensitivity analyses yielded the same inferential pattern for relocation category in Model 1, F(2,88) = 9.87, *p* < 0.001, and Model 2, F(2,79) = 9.03, *p* < 0.001.

The association between relocation category and loneliness was weaker in the extended complete-case model, but Model 1 and the extended model used different analytic samples. To distinguish covariate adjustment from sample composition, Model 1 was re-estimated in the same 76 participants included in the extended model. In this restricted Model 1, the overall relocation-category term remained statistically significant, F(2,70) = 3.88, *p* = 0.025. Relative to one relocation, the coefficient for two to three relocations was B = 4.749 (95% CI [0.830, 8.668], *p* = 0.018), and the coefficient for four or more relocations was B = 6.245 (95% CI [1.003, 11.486], *p* = 0.020). In the extended model, the corresponding coefficients were B = 1.933 (95% CI [−1.323, 5.189], *p* = 0.240) and B = 3.215 (95% CI [−1.086, 7.516], *p* = 0.140), respectively. In the extended complete-case model that additionally included depressive symptoms, current physical health, and household income (*n* = 76), the full model was significant, F(8,67) = 11.29, *p* < 0.001, R^2^ = 0.574, but the overall relocation-category term was no longer statistically significant, F(2,67) = 1.23, *p* = 0.298. Depressive symptoms (B = 1.095, *p* < 0.001), higher household income (B = −1.592 per income category, *p* = 0.011), older age (B = 0.240, *p* = 0.020), and having a partner (B = −3.232, *p* = 0.041) were associated with loneliness in this model; current physical health and gender were not. A further complete-case sensitivity analysis adding reported years of education (n = 59) yielded the same conclusion for relocation category (overall F(2,49) = 1.42, *p* = 0.251); the full model was significant, F(9,49) = 10.90, *p* < 0.001, R^2^ = 0.667. Using HC3 robust standard errors, the relocation-category term in the extended model also remained non-significant, F(2,67) = 1.69, *p* = 0.191. Treating household income categorically rather than as an ordered linear predictor likewise yielded a non-significant relocation term, F(2,64) = 1.33, *p* = 0.271. Because both relocation coefficients were smaller in the extended model and their 95% confidence intervals shifted to include zero within the identical 76-participant sample, the attenuation cannot be attributed solely to sample composition. The comparison should therefore be interpreted in terms of coefficient magnitude and confidence intervals rather than the change in statistical significance alone. An additional sensitivity analysis that retained the 26 respondents selecting “no relocation” as a fourth response category yielded the same qualitative pattern: the overall relocation-category association was statistically significant in the demographic-adjusted model (*p* < 0.001) but not in the extended model (*p* = 0.293) (Table 3).

**Table 3:** Regression coefficients for models predicting loneliness from relocation-frequency category and covariates.

Panel A—Demographic-adjusted model (N = 94)—overall relocation-frequency term: F(2,88) = 7.40, *p* = 0.001, partial R^2^ = 0.144.						
**Predictor**		**B**	**SE**	**95% CI**	**t**	***p***
2–3 relocations vs. one		5.959	1.665	[2.650, 9.268]	3.579	<0.001
≥4 relocations vs. one		6.499	2.251	[2.025, 10.973]	2.887	0.005
Age		0.349	0.114	[0.123, 0.576]	3.064	0.003
Gender (female vs. male)		−0.0492	1.749	[−3.968, 2.983]	−0.282	0.779
With partner vs. without		−4.505	1.669	[−7.821, −1.189]	−2.700	0.008
Panel B—Model additionally including perceived community resilience (N = 86)—overall relocation-frequency term: F(2,79) = 7.19, *p* = 0.001, partial R^2^ = 0.154.						
**Predictor**		**B**	**SE**	**95% CI**	**t**	***p***
2–3 relocations vs. one		6.071	1.679	[2.729, 9.412]	3.616	<0.001
≥4 relocations vs. one		6.095	2.261	[1.594, 10.596]	2.695	0.009
Community resilience		−0.312	0.099	[−0.510, −0.114]	−3.143	0.002
Age		0.324	0.114	[0.098, 0.550]	2.849	0.006
Gender (female vs. male)		−0.897	1.744	[−4.369, 2.575]	−0.514	0.609
With partner vs. without		−4.609	1.694	[−7.982, −1.237]	−2.720	0.008
Panel C—Extended sensitivity model based on Model 1, additionally adjusting for depressive symptoms, current physical health, and household income (N = 76)—overall relocation-frequency term: F(2,67) = 1.23, *p* = 0.298, partial R^2^ = 0.036.						
**Predictor**		**B**	**SE**	**95% CI**	**t**	***p***
2–3 relocations vs. one		1.933	1.631	[−1.323, 5.189]	1.185	0.240
≥4 relocations vs. one		3.215	2.155	[−1.086, 7.516]	1.492	0.140
Age		0.240	0.101	[0.038, 0.442]	2.374	0.020
Gender (female vs. male)		−0.708	1.657	[−4.016, 2.600]	−0.427	0.671
With partner vs. without		−3.232	1.553	[−6.331, −0.132]	−2.081	0.041
Depressive symptoms (GDS-15)		1.095	0.296	[0.504, 1.685]	3.700	<0.001
Current physical health		−0.933	0.868	[−2.666, 0.800]	−1.074	0.286
Household income		−1.592	0.606	[−2.801, −0.383]	−2.629	0.011
Panel D—Same-sample comparison of Model 1 and the extended sensitivity model (N = 76).						
**Model**	**Contrast**	**B**		**95% CI**		***p***
Model 1 (same sample)	2–3 relocations vs. one	4.749		[0.830, 8.668]		0.018
	≥4 relocations vs. one	6.245		[1.003, 11.486]		0.020
Extended sensitivity model	2–3 relocations vs. one	1.933		[−1.323, 5.189]		0.240
	≥4 relocations vs. one	3.215		[−1.086, 7.516]		0.140

Overall relocation-frequency term: Model 1, F(2,70) = 3.88, *p* = 0.025; extended sensitivity model, F(2,67) = 1.23, *p* = 0.298. Notes: Relocation frequency was indicator-coded with “one relocation” as the reference category. Gender was coded female versus male; the single participant selecting another gender category was retained in descriptive analyses but omitted from regression models requiring binary gender coding. Partner status was coded with partner versus without partner. Coefficients are unstandardized.

## 5. Discussion

The present study examined post-return loneliness across self-reported relocation-frequency categories and its concurrent association with perceived community resilience among older evacuees. Three findings stand out. Loneliness differed across relocation categories after demographic adjustment, but the pattern was not monotonic; both higher-frequency groups reported more loneliness than the one-relocation group. Higher perceived community resilience was associated with lower loneliness. Finally, in the same 76 participants, relocation coefficients were attenuated after additional adjustment for depressive symptoms, physical health, and household income. Together, the results suggest that post-return loneliness reflects relocation experiences in conjunction with community and individual circumstances rather than relocation frequency alone.

From an environmental gerontology perspective, evacuation is better understood as a process of environmental discontinuity than as a simple transition from home to displacement. Place in later life encompasses routines, social networks, services, and symbolic attachments [3,4,5,29,30]; repeated moves may disrupt several of these simultaneously. The observed heterogeneity among evacuees therefore extends the focus beyond whether evacuation occurred to how residential transitions were experienced.

The non-monotonic pattern also argues against a simple dose–response interpretation. Two to three relocations were associated with the highest unadjusted loneliness, whereas four or more relocations did not produce a further increase. Moves may differ in duration, destination, social context, and disruption of routines or support, none of which were measured here. Because relocation frequency was captured by a single item without explicit counting instructions, the coefficients should be interpreted as associations with self-reported categories rather than precise effects of the number of moves.

The same-sample comparison further qualifies the relocation finding. After depressive symptoms, physical health, and income were added, both relocation coefficients decreased and their confidence intervals included zero, indicating that the attenuation was not solely a consequence of the smaller analytic sample. This is consistent with a person–environment perspective in which environmental disruption is intertwined with health, socioeconomic, and relational resources [3]. Because these covariates were measured after return, however, the analysis cannot distinguish confounding from possible overadjustment for factors that may themselves have been influenced by evacuation.

Perceived community resilience showed a clearer concurrent association with loneliness. Participants who viewed their permanent/home community as more resilient reported lower loneliness, linking the findings to current discussions of inclusive and resilient places for ageing [15,16,50,52]. The measure referred to the home community after return; so, it does not show that resilience in temporary evacuation settings protected against loneliness during displacement. It nevertheless indicates that the social and institutional qualities of the home community remain relevant after forced displacement.

This finding also broadens the meaning of ageing in place under crisis conditions. When remaining physically at home becomes temporarily impossible, continuity may depend on maintaining social, institutional, and symbolic connections to place rather than physical continuity alone.

A resilient place for ageing may therefore extend beyond the dwelling itself to relationships with neighbors, local leadership, municipal services, and familiar health and welfare providers. Preserving such connections across temporary settings is a plausible direction for future research, but these mechanisms were not directly measured in the present study.

Relocation category was not significantly associated with community resilience when community resilience was modeled as the outcome. The data therefore do not support a mediation interpretation; perceived community resilience is best treated here as a concurrent correlate of loneliness. Because it was assessed after return, this study cannot determine how community conditions during displacement were related to relocation history or loneliness.

Age and partner status also mattered: older age and not having a partner were associated with higher loneliness, whereas gender was not. These findings reinforce the need to avoid treating older evacuees as a homogeneous group and to consider differences in social support and individual resources [18,20,64].

Similarly, the extended analysis linked loneliness with depressive symptoms and household income. Taken together, the findings fit a transactional environmental-gerontology perspective in which post-return well-being emerges from the interaction between environmental disruption, community resources, and individual circumstances. The cross-sectional design does not establish how these factors operated during evacuation, but the findings support a more dynamic understanding of ageing in place when continuity of place is interrupted.

### 5.1. Implications

The findings support a concise set of practice implications. Relocation history may be useful as one component of psychosocial assessment after evacuation, but it should not be used as a stand-alone risk marker. Assessment should also consider loneliness, depressive symptoms, partner and family circumstances, health, socioeconomic resources, and perceived community support.

For evacuation planning, the results are most consistent with preserving continuity rather than with any specific intervention tested here. When feasible, temporary arrangements could seek to maintain access to familiar services, community contacts, and social networks while also supporting accessibility, privacy, and autonomy. Because these features were not measured during evacuation, they should be evaluated in longitudinal and intervention studies rather than treated as demonstrated effects.

Temporary accommodation should therefore be viewed not only as a site of physical safety but also as an environment in which older adults must maintain everyday functioning. This may be particularly important for people with fewer social, health, or economic resources.

More broadly, ageing-in-place planning for emergencies should include strategies for maintaining social, institutional, and symbolic continuity when physical residence in place is temporarily impossible.

### 5.2. Limitations

Several limitations should be considered. First, the cross-sectional design precludes conclusions about temporal ordering or causality because loneliness, community resilience, and covariates were measured after participants returned home. The findings therefore describe post-return associations, and longitudinal data are needed to examine change across evacuation and return.

Second, relocation frequency was measured with a single ordinal item whose counting rule was not stated explicitly. Participants may have differed in whether they counted the initial evacuation or subsequent moves, and the 26 respondents selecting “no relocation” had nevertheless evacuated. Their exclusion from the primary analysis therefore introduces classification uncertainty, although retaining them as a fourth category produced the same qualitative pattern. The item also did not capture duration, voluntariness, distance, accommodation quality, social context, or perceived disruption and should not be interpreted as a comprehensive measure of relocation instability.

Third, this study relied on self-report, and perceived community resilience referred only to the permanent/home community after return rather than to temporary settings. The sample came from one community and geopolitical context, used non-probability recruitment, and was restricted to returnees; a response rate could not be calculated. These features create potential selection bias and limit generalizability to older evacuees who remained displaced, relocated permanently, or were otherwise not represented. Missing data further reduced the regression samples, and complete-case selection may have been non-random, particularly with respect to partnership status. The age-50-plus recruitment framework also limits generalization across older populations, although the age-65-plus sensitivity analysis produced the same overall conclusion for relocation category. Finally, residual confounding remains possible because several relevant factors were not assessed, and post-return measurement of depressive symptoms and physical health means that their role as confounders, correlates, or consequences cannot be determined.

## 6. Conclusions

Despite these limitations, this study highlights the value of examining evacuation through an environmental gerontology lens. The findings show heterogeneity in post-return loneliness across self-reported relocation-frequency categories in demographic-adjusted analyses and a concurrent association between current perceived resilience of the permanent/home community and lower loneliness. The attenuation of the relocation association after broader adjustment also indicates that environmental experiences should be considered together with mental-health, socioeconomic, and relational circumstances. This perspective may help broaden research on ageing in place toward a more dynamic understanding of how supportive and resilient environments can be maintained when older adults are temporarily displaced.

## Figures and Tables

**Table 1:** Descriptive statistics of the study variables (N = 109; valid N varies by variable because of item nonresponse).

Variable	Category	N (Valid %)	Mean (SD)	Range
Gender	Female	84 (77.1)	—	—
	Male	24 (22.0)	—	—
Age	—	102	69.5 (7.0)	54–89
Partnership status	With partner	65 (60.7)	—	—
	Without partner	42 (39.3)	—	—
Loneliness	—	102	25.1 (8.0)	11–45
Community resilience	—	96	34.3 (7.8)	17–50
Relocation frequency	One relocation	32 (29.4)	—	—
	2–3 relocations	58 (53.2)	—	—
	≥4 relocations	19 (17.4)	—	—

Note: One participant (0.9%) selected another gender category and is included in the analytic sample.

**Table 2:** Bivariate associations between study variables and loneliness (N = 109; test-specific N varies because of item nonresponse).

Variable	Statistical Test	Statistic	*p*
Gender (male vs. female)	Independent-sample *t* test	t = 0.52	0.604
Age	Pearson’s correlation	r = 0.32	0.001
Partner status	Independent-sample *t* test	t = −4.00	<0.001
Relocation-frequency category	One-way ANOVA	F = 5.07; η^2^ = 0.093	0.008
Community resilience	Pearson’s correlation	r = −0.39	<0.001

## Data Availability

The data are not publicly available due to privacy and confidentiality restrictions.
